# Supplementary material for: Dynamic residue interaction network analysis of the oseltamivir binding site of N1 neuraminidase and its H274Y mutation site conferring drug resistance in influenza A virus
Source: PeerJ. 2021 Jun 2;9:e11552. doi: 10.7717/peerj.11552 (PMC8179223; doi:10.7717/peerj.11552)
Supplement: Supplemental Information 1 — Binding free energy consists of van der Waals (vdW), electrostatic (ES), polar solvation, and non-polar solvation components. [file peerj-09-11552-s001.docx]

|  | vdW  [kcal mol^−1^] | ES  [kcal mol^−1^] | polar  [kcal mol^−1^] | non-polar  [kcal mol^−1^] | total  [kcal mol^−1^] |
| --- | --- | --- | --- | --- | --- |
| WT | −28.19 ± 0.08 | −46.96 ± 0.08 | 43.51 ± 0.06 | −3.54 ± 0.00 | −35.19 ± 0.07 |
| H274Y | −22.50 ± 0.09 | −40.82 ± 0.10 | 39.35 ± 0.08 | −3.31 ± 0.00 | −27.28 ± 0.09 |
