# Supplementary material for: Dynamic residue interaction network analysis of the oseltamivir binding site of N1 neuraminidase and its H274Y mutation site conferring drug resistance in influenza A virus
Source: PeerJ. 2021 Jun 2;9:e11552. doi: 10.7717/peerj.11552 (PMC8179223; doi:10.7717/peerj.11552)
Supplement: Supplemental Information 2 [file peerj-09-11552-s002.docx]

|  | Δ*H*  [kcal mol^−1^] | *T*Δ*S*  [kcal mol^−1^] | Δ*G*  [kcal mol^−1^] | ΔΔ*G*  [kcal mol^−1^] |
| --- | --- | --- | --- | --- |
| WT | −32.84 ± 0.13 | −23.65 ± 0.47 | −9.19 ± 0.49 |  |
| H274Y | −21.65 ± 0.19 | −22.94 ± 0.50 | 1.29 ± 0.53 | 10.48 |
